# Establishment of resilience in a challenging recovery at home after pediatric tonsil surgery—Children’s and caregivers’ perspectives

**DOI:** 10.1002/pne2.12051

**Published:** 2021-05-10

**Authors:** Fredrik Alm, Gunilla Lööf, Karin Blomberg, Elisabeth Ericsson

**Affiliations:** ^1^ Department of Anaesthesia and Intensive Care School of Health Sciences Faculty of Medicine and Health Örebro University Örebro Sweden; ^2^ Department of Paediatric Anaesthesia and Intensive Care Astrid Lindgren Children’s Hospital Karolinska University Hospital Stockholm Sweden; ^3^ School of Health Sciences Faculty of Medicine and Health Örebro University Örebro Sweden

**Keywords:** caregivers, child, postoperative pain management, recovery, tonsil surgery

## Abstract

The objective of this study was to explore children's and caregivers’ experiences and management of postoperative recovery at home after tonsil surgery. The study had an explorative qualitative design with an inductive approach. Twenty children (5‐12 years of age) undergoing tonsillectomy or tonsillotomy with or without adenoidectomy participated along with their caregivers in semi‐structured interviews at a mean time of 28 days after surgery. The interviews were analyzed with content analysis. One main category emerged from the interviews: children and caregivers struggle to establish resilience in a challenging recovery. The families’ resilience relied on their situational awareness and capacity to act, which in turn formed a basis for the ability to return to normal daily life. Children and caregivers described the recovery as an evident interruption of daily life which had an impact on the children's physical and psychological well‐being. Both children and caregivers described the pain as a central concern. The families used different pharmacological and complementary strategies to manage the pain, which in some cases were complex. Some families said that the analgesics were insufficient in preventing breakthrough pain, and spoke about a lack of support as well as inadequate and contradictory information from healthcare staff. Caregivers also expressed uncertainty, ambivalence, or anxiety about the responsibility associated with their child's recovery. To optimize and support the recovery after tonsil surgery, it is crucial to obtain knowledge of children's and caregivers’ perspectives of postoperative recovery at home. The results indicate that the postoperative period included several troublesome experiences for which neither the children nor the caregivers were informed or prepared. The experience of pain was significant, and often complex to manage. To increase families’ resilience, the information provided by healthcare professionals needs to be broadened. Multidisciplinary teamwork is necessary to achieve this goal.

## 1 INTRODUCTION

Tonsil surgery is one of the most common surgical procedures in children worldwide. The primary indications are upper airway obstruction related to tonsillar hypertrophy and recurrent tonsillitis. Although tonsil surgery is associated with health benefits,^1^, ^2^ a child undergoing tonsil surgery is emotionally and physically affected throughout the process, not least postoperatively affected by pain and pain‐related complications.^3^, ^4^

There are two surgical methods in tonsil surgery practice: tonsillectomy and tonsillotomy. In tonsillectomy, the entire tonsil is removed, including its capsule, while tonsillotomy involves surgical removal of the majority of the tonsillar tissue without violating the capsule. Tonsillectomy continues to be the predominant method regarding infection‐related indications, while tonsillotomy, in some countries, has become the most common method for indication of obstruction.^5^, ^6^ The advantages of tonsillotomy over tonsillectomy are that it reduces postoperative pain, the need for analgesics, and the risk of postoperative hemorrhage while producing faster recovery and a similar level of symptom relief.^7^, ^8^, ^9^, ^10^, ^11^, ^12^ Since high pain levels are reported after both methods,^4^ it is essential to ensure adequate pain management for all patients undergoing tonsil surgery.

Tonsil surgery is often performed in outpatient settings,^13^ meaning that the hospitalization is short and most of the recovery takes place at home. This puts significant demands on families to manage varied conditions.^14^, ^15^ A central concern is the postoperative pain and pain management, as the families are required to make accurate assessments and administer the correct analgesic doses along with non‐pharmacological strategies.^16^ Despite a growing body of evidence highlighting the importance of appropriate doses and regular administration to achieve analgesia,^17^ studies indicate that healthcare fails to transmit these aspects to the home setting.^18^, ^19^

Knowledge of children's and caregivers’ perspectives is needed in order to optimize and support recovery after tonsil surgery. In the research, the difference between taking the child's perspective and having a child perspective needs to be recognized. A child perspective is based on the adult's outside perspective on children's conditions, experiences, perceptions, and actions, while the child's perspective includes the child's insider perspective on these conditions, experiences, perceptions, and actions.^20^ Even though children are competent in describing their discomfort and should be viewed as experts in their own recovery,^21^ caregivers’ supportive and decision‐making perspectives are also essential in the evaluation of children's recovery after tonsil surgery. Thus, investigating children's and caregivers’ perspectives of the postoperative period at home after tonsil surgery is crucial in attempting to understand and evaluate healthcare interventions and thereby improve the effectiveness and safety of the service provided. This study aimed to explore children's and caregivers’ experiences and management of postoperative recovery at home after tonsil surgery.

## 2 METHODS

### 2.1 Design

An explorative qualitative design with an inductive approach was chosen.^22^ Qualitative methods aim to understand a complex reality and the meaning of actions in a given context. In addition, a qualitative approach preserves the child's perspective, allowing them to highlight what they think is important. Thus, a qualitative design is appropriate when the objective is to obtain an in‐depth understanding of experiences.

### 2.2 Sample and setting

The participants consisted of children (aged 5‐12 years) undergoing elective tonsil surgery (tonsillectomy or tonsillotomy with or without adenoidectomy) and their caregivers. Exclusion criteria were the child and/or caregiver's inability to speak and/or understand Swedish, and the presence of cognitive disorders or physical conditions in the child that interfered with the standard care program.

Participants were selected from a cohort (aged 4‐17 years) included in an observational study that used a pain diary to explore postoperative pain, pain management, and recovery at home after tonsil surgery.^4^ Families from three hospitals were informed of the possibility to participate in an interview 2‐4 weeks after surgery. After returning the pain diary, 20 children and their caregivers were purposively selected from the cohort to include a variety of gender, ages, and surgical methods. The families were contacted via phone by the first author (FA) and given the choice of when and where the interviews were to be conducted.

### 2.3 Data collection

Semi‐structured interviews were conducted by the first author and followed a pilot‐tested interview guide including opening and probing questions (Appendix 1 ). The interview technique was flexible, and the questions were modified to be compatible with the individual child's cognitive and linguistic stage of development. All interviews were audio‐recorded and lasted 20‐45 minutes, with most of the time consumed by the child´s interview. Interviews were performed in the child's home (n = 18), at the library (n = 1), and at a café (n = 1), an average of 28 days after surgery (range: 14‐43 days).

The child was interviewed first, with the caregiver present, and this was followed by the interview with the caregiver(s). In situations when a caregiver influenced the interview with the child, the interviewer returned the focus of the interview to the child and the caregiver's input was used as a resource to proceed with the interview; for example, “Your mother/dad says… can you describe this for me?”

To establish a relaxed atmosphere, the interviews with the children started with an informal conversation about interests and daily activities. The conversation continued with the main opening question for the interview: “Can you tell me about the days at home after your tonsil surgery?” The child's answer governed the continued direction of the interview. The pre‐filled diary constituted support to help the child remember and understand the context for the interview.

Probing questions such as “You said pain; can you tell me about a situation when you were in pain?” or “When it happened, what did you do?” were used to get a deeper understanding and to capture the children's own perspectives and management of the situation. The children's limited ability to understand abstract concepts was taken into consideration. When inconsistencies were identified in a child's answers, a reflective interview technique was used to check what the child really meant or said.^23^ In cases where the child struggled to answer or to recall the days after surgery, supportive questions such as “What did you eat after returning home from the hospital?” were used to help them remember.

The main opening question in the caregiver interviews was “Can you tell me about the days at home after your surgery?” Caregivers’ answers were followed up with the same type of probing questions as in the interviews with children. Other questions in these interviews were formulated on the basis of findings from the child's interview, in order to capture caregivers’ perspectives on the same phenomena. No new information was obtained in the last few interviews, and it was judged that data saturation had been reached.

### 2.4 Analysis

All interviews were transcribed verbatim by the first author (FA: n = 1) and a professional transcriber (n = 19). The analysis was guided by inductive content analysis^22^ and primarily conducted by the first author together with the research team, representing knowledge within the areas of anesthesia, pediatrics, otorhinolaryngology, and qualitative methodology. The transcripts were first to read independently by all authors to familiarize themselves with the data. Meaning units were identified, condensed, and labeled with codes via notes and comments in the margins. The codes were then sorted in a familiar specific coding sheet and grouped into ten subcategories. Finally, the latent level of the categories was interpreted. Three categories and one main category were generated through discussions and consensus in the research team.

Although the children's and caregivers’ interviews were separately handled and analyzed throughout the analysis process, data from both groups could be classified under the same main category. The research team continuously returned to the transcribed data to ensure that the analysis and the coding accurately reflected what the children and caregivers had said, and to confirm the consistency of the main category. To ensure trustworthiness, methodological considerations were taken into account in relation to the principles described by Lincoln and Guba.^24^ For example, the analytical process was guided by several discussions between the researchers in order to ensure confirmability, and quotations were included in the presentation of the results in order to increase the credibility.

### 2.5 Ethics

Ethical approval was obtained from the Regional Ethical Review Board, Uppsala, Sweden (no. 2017/169). Age‐differentiated information (written and verbal) was provided to both caregivers and children, and all interviewees provided their signed consent to participate in both studies. Developmental limitations and the imbalance of power between children, caregivers, and healthcare providers were taken into consideration throughout the research process. Both children and caregivers were informed that consent was optional and possible to withdraw at any point.

## 3 RESULTS

The children were all aged 5‐12 years and had undergone tonsillectomy (n = 14) or tonsillotomy (n = 6). Fifteen children were interviewed with the mother, one child with the father, and four children with both the mother and the father. The children were pain‐free after a median of 8 days. One child had previous experience of surgery (adenoidectomy). The profile of the participants is described in Table 1.

**TABLE 1:** Children's age, sex, surgical method/indication, pain score, and relation to the interviewed caregiver(s)

Family	Child age	Sex	Surgical method	Main surgical indication^a^	First pain‐free day	Number of days with FPS‐R score ≥4	Interviewed caregiver(s)
1	9	Girl	TE	Obstruction	10	7	Mother
2	12	Boy	TE	Infection	8	5	Mother + father
3	10	Boy	TE	Obstruction	9	6	Mother
4	5	Boy	TEA	Obstruction	5	4	Mother
5	5	Girl	TEA	Obstruction	9	4	Mother
6	8	Boy	TT	Obstruction	6	3	Mother
7	7	Girl	TEA	Obstruction	8	2	Mother
8	8	Boy	TEA	Obstruction	10	8	Mother
9	6	Girl	TEA	Obstruction	>12	>12	Mother
10	10	Girl	TTA	Obstruction	4	3	Mother
11	9	Girl	TE	Infection	7	5	Mother +father
12	12	Boy	TE	Infection	11	6	Father
13	10	Boy	TE	Infection	9	7	Mother
14	11	Boy	TE	Infection	11	5	Mother + father
15	6	Boy	TTA	Obstruction	7	7	Mother
16	8	Boy	TTA	Obstruction	9	7	Mother
17	5	Boy	TE	Obstruction	11	6	Mother
18	5	Boy	TTA	Obstruction	7	3	Mother
19	5	Girl	TTA	Obstruction	4	3	Mother + father
20	6	Girl	TE	Obstruction	4	3	Mother

Abbreviations: FPS‐R, Faces Pain Scale — Revised;TE, tonsillectomy; TEA, tonsillectomy with adenoidectomy; TT, tonsillotomy; TTA, tonsillotomy with adenoidectomy.

^a^ Obstruction = upper airway obstruction, Infection = recurrent tonsillitis/chronic tonsillitis.

One main category emerged from the interviews: children and caregivers struggle to establish resilience in a challenging recovery. Resilience refers to a family's ability to withstand and recover from the disruptions involved in tonsil surgery. The families’ resilience relied on their situational awareness and capacity to act, which in turn formed a basis for managing the impact on daily life and ability to return to normal daily life. The main category rested on three categories: situational awareness, capacity to act, and impact on daily life, which in turn were based on ten subcategories including data from both children and caregivers (Table 2). Quotations from the children and caregivers organized in terms of subcategories associated with the three categories are presented in Tables 3, 4, 5.

**TABLE 2:** The main category, categories, subcategories, and data sources

Main category	Categories	Subcategories	Data source
Children and caregivers struggle to establish resilience in a challenging recovery	Situational awareness	Information and instructions	Children and caregivers
		Frames of reference	Children and caregivers
		Observation and comprehension	Children and caregivers
	Capacity to act	Pharmacological strategies	Children and caregivers
		Complementary strategies	Children and caregivers
		Compliance	Children and caregivers
		Self‐reliance	Caregivers
	Impact on daily life	Physical reactions	Children and caregivers
		Psychological and behavioral reactions	Children and caregivers
		Impact on basic functions	Children and caregivers

**TABLE 3:** Quotations from children and caregivers, organized by subcategories associated with the category “situational awareness”

Subcategory	Children	Caregivers
Information and instruction	“I wasn't allowed to be physically active because it could start to bleed in my mouth.” [Child, family 8] “The doctor said I was not allowed to move around at all in two weeks.” [Child, family 11]	“We wondered, can he be active now? Should he exercise? We didn't know. We thought it was a bit unclear.” [Caregiver, family 6] “At the hospital, they said to take medicine according to the schedule for three days, but she didn't get better. Instead, she complained even more. So I continued with analgesics.” [Caregiver, family 5] “The pharmacist said no, no, no, the doctor has prescribed too high a dose, this dose can harm her. We got worried and we had to call the hospital again.” [Caregiver, family 19] “We slept in the same room due to the risk of bleeding and being close to the child.” [Caregiver, family 1]
Frames of reference	“The discomfort (after surgery) was quite similar to when I had a throat infection.” [Child, family 2] “After using melting tablets which I thought was disgusting we bought regular ones which worked out quite well. I had learned how to swallow tablets during my previous tonsillitis.” [Child, family 12]	“He has had surgery once before, about a year ago, an adenoidectomy. So, we kind of expected it to be pretty similar, but it wasn't… we really got to notice the difference.” [Caregiver, family 15] “Big sister has undergone the same operation. Two things separate their recovery: the difficult time after the operation and the bleeding. So, I’m wondering if they might used a different method?” [Caregiver, family 3]
Observation and comprehension	“It got better (after taking medicine). It was as normal for a while, but later the pain crept in again.” [Child, family 6] “The neck and ears are connected with nerves so they (ears) hurt too.” [Child, family 1]	“The pain was worst in the morning, because we didn't wake him (for analgesic administration).” [Caregiver, family 2] “As long as analgesics were given within six hours, it was great. She could run around as if nothing had happened.” [Caregiver, family 20] “When a long time had passed between the first and second dose, she lay on the sofa, red in the face, completely powerless.” [Child, family 20]

**TABLE 4:** Quotations from children and caregivers, organized by subcategories associated with the category “capacity to act”

Subcategory	Children	Caregivers
Pharmacological strategies	“I always took juice or sparkling water after (taking the analgesics), or we mixed the medicine with water.” [Child, family 7] “It was easiest to take the medicine together with chocolate pudding.” [Child, family 8]	“When we talked about analgesics, he wanted a mixture, but it turned out that he found them disgusting. So, we needed to recalculate the dosage. We finally had to call and ask healthcare; we were a little unsure of the doses.” [Caregiver, family 6] We had practiced swallowing candies similar to the tablets but, due to severe pain we had to use suppositories the first days of recovery. As soon as it worked to swallow again, we gradually started using split tablets mixed with soft ice cream. [Caregiver, family 1]
Complementary strategies	“When I was in a lot of pain, I just lay on the couch and ate ice cream because it slid down so easily in my throat.” [Child, family 1] “Chewing gum was good because it created saliva, which made it less painful.” [Child, family 12]	“So we lay together on the couch, stroking her back and hair, talking about funny things, and watching good movies. Just to be able to focus on something together, to focus away the pain.” [Caregiver, family 1] “There wasn't much to do except give painkillers, be there and give support and comfort, give ice cream, and do something to forget the pain, like play cards or something” [Caregiver, family 2]
Compliance	“It tasted terrible (analgesics), but I swallowed it anyway.” [Child, family 6] “the first week I was not allowed to run and play, so I took it easy.” [Child, family]	“We celebrated Easter quietly; I thought she wouldn't have been able to sit at dinner and hang out with relatives.” [Caregiver, family 5] “The prescription we received was to give medicine every 6 hours, around the clock, for three days. So, we stopped (after three days). But it didn't work, and we had to continue the medication. He was having a difficult time before we started (the medicine) again.” [Caregiver, family 3]
Self‐reliance		“I had a hundred thoughts. Should I call or go (to the health service)? No, we won't go, we'll just hope we survive the night.” [Caregiver, family 9] “We gave too high a dose of ibuprofen, 400mg instead of 200mg, but it wasn't a problem because we solved it by waiting longer before we gave the next dose.” [Caregiver, family 2]

**TABLE 5:** Quotations from children and caregivers, organized by subcategories associated with the category “impact on daily life”

Subcategory	Children	Caregivers
Physical reactions	“The pain was just like scraping your knee, but in your throat.” [Child, family 6] “It was very, very, very painful to swallow and eat.” [Child, family 9]	“There was a setback (after a few days). It got difficult again. It was as if the recovery started again from the beginning.” [Caregiver, family 7] “He doesn't usually complain, but then he cried and screamed.” [Caregiver, family 17]
Psychological and behavioral reactions	“I didn't play because I was afraid that something would happen (that it would start to bleed).” [Child, family 3] “I’m a bit scared because I don't like to take tablets, because I have a very hard time swallowing them.” [Child, family 1]	“All week he didn't want to be alone when he was sleeping. He was scared. He lay over my stomach and legs all night. He needed to have body contact with me all the time. He had nightmares and was restless in his body.” [Caregiver, family 15] “The first three nights went well, then he started to have night terrors. He screamed and we couldn't get through to him. It went on, maybe once an hour.” [Caregiver, family 4] “He was tired and sad, as if he were depressed. I didn't see the usual twinkle in his eye.” [Caregiver, family 8] “I slept with her all the time with us. She was so anxious.” [Caregiver, family 9]
Impact on basic functions	“I just lay on the couch and rested because I couldn't do anything, talk or anything; it was hard.” [Child, family 12] “I could only play a little bit.” [Child, family 9] “I wasn't allowed to get up and move or anything like that. We bought cream because I couldn't eat and swallow anything else.” [Child, family 11]	“For three days, he lived on ice water and ibuprofen.” [Caregiver, family 11] “First three days he was completely powerless and he was just lying on the couch; we had to carry him around, he was so tired. Very restless sleep and a lot of nightmares.” [Caregiver, family 15]

### 3.1 Situational awareness

This category refers to the children's and caregivers’ situational awareness, influenced by inadequate and contradictory information, reference frames from family and friends and previous experiences, and the ability to observe and comprehend the situation. The category is merged into three subcategories: information and instructions, frames of reference, and observation and comprehension (Table 3).

#### 3.1.1 Information and instructions

The caregivers expressed unpreparedness and described several situations in which they would have needed help and guidance during their child's recovery. Although the need for information was generally described as being met, some caregivers found the information received to be inadequate and contradictory. Information associated with pain management was highlighted in terms of discrepancies between the duration of the analgesic administration in the dosing schedule delivered and the child's need for pain relief. This resulted in complicated breakthrough pain and a need for caregivers to extend the analgesic treatment. Furthermore, the information and instructions about pain management varied between different health professionals and between hospitals and pharmacies, making caregivers both confused and worried about harming their child.

Both children and caregivers described an awareness and fear of the risk of postoperative bleeding based on the information given. Many caregivers slept together with their child in order to control the risk of bleeding, and restriction of physical activities was described by both parties. Despite children's and caregivers’ compliance with this important information, many experienced a lack of clarification regarding the level of physical activity permitted, meaning that most of the children were inactive and stayed inside the house during most of their recovery.

#### 3.1.2 Frames of reference

Data from both children and caregivers clearly indicated the need to establish frames of reference to which the postoperative experiences could be related. Caregivers related their child's recovery to previous operations and their own or someone else's tonsil surgery (the grandmother or sibling of the child, or a colleague's child). The differences between positive and negative outcomes of perioperative care were energetically argued, and caregivers speculated about various surgical methods or characteristics of the child as underlying reasons for the outcome. Children's postoperative discomfort was mainly related to a frame of reference associated with previous experiences of tonsil infections described in terms of difficulties with pain and swallowing.

#### 3.1.3 Observation and comprehension

Children described varied comprehension of the observations associated with their recovery. A few were able to situate the individual symptoms in a broader perspective and understand their significance in the context of the postoperative recovery, such as why earache was associated with tonsil surgery. As the postoperative days went on, the children established strategies to manage the situation based on their own experiences and were increasingly able to point out the source of discomfort and suggest preventive actions such as avoiding certain types of food or taking analgesics.

Caregivers’ comprehension of their observations differed widely between the families. The caregivers described how they established an understanding of the connection between their child's pain and analgesic administration. By monitoring changes in the child's mood, energy level, and ability to concentrate, the caregivers were able to see the effect of the analgesics. This often resulted in an adjustment to a more regular administration of analgesics, producing an optimized pain treatment. Another common area of concern and lack of understanding described by caregivers was the new onset of sleep disturbance, awakenings, and nightmares in their child after being discharged from the hospital.

### 3.2 Capacity to act

This category refers to the children's and caregivers’ capacity to act by using different strategies, by adapting to the prevailing circumstances, and via self‐reliance. The category is merged into four subcategories: pharmacological strategies, complementary strategies, compliance, and self‐reliance (Table 4).

#### 3.2.1 Pharmacological strategies

Pain medication was a significant part of the children's relief strategy during their postoperative recovery. Some children considered themselves pain‐free after taking analgesics, while others described insufficient pain relief. The children expressed a wide spectrum of difficulties associated with medicine, including the painfulness of swallowing the tablets and the medicine's bad taste and sticky consistency. They also shared several innovative procedures to facilitate the analgesic intake, such as always drinking something immediately afterward or mixing the analgesic with a certain type of food (for example yogurt, water or chocolate mousse).

Some caregivers considered analgesics to be insufficient due to their slow onset and suboptimal effect in terms of avoiding breakthrough pain. When paracetamol and ibuprofen were insufficient, the caregivers lacked both instructions and analgesics potent enough to handle the pain at its worst. Many caregivers also described reactions such as powerlessness, listlessness, panicking and crying due to insufficient or delayed analgesic administration. Some caregivers emphasized the importance of regular administration to achieve adequate pain relief. Setting an alarm and keeping a logbook were frequently used to establish structure in an around‐the‐clock regimen. Many caregivers described how they alternated both the formulation and route of the analgesics during the recovery, such as using suppositories during the night as an attempt to facilitate the analgesic administration.

The caregivers discussed multiple problems with the management of the analgesics, from the pharmacies not stocking the right strength or formulation to the child refusing to take the analgesics. To cope with the situation, caregivers were often forced to change the prescribed formulations and/or route. Several of them described this as challenging, since it necessitated repeated telephone contacts with healthcare staff and some raised concerns associated with the risk of incorrect dosing in connection with a change of the primary prescription.

#### 3.2.2 Complementary strategies

The children described the use of various complementary strategies to manage their recovery, including physical and cognitive‐behavioral methods. Ice cream and other types of alleviative food (eating and drinking cold foods and fluids, as well as ice) were often mentioned as pain relief. Several children said that chewing gum was comforting, both regarding increased salivation and as a starter to make it easier to chew their food. To get relief from the earache, one child found it helpful to press and rub their hands against their ears. Several children reported “relaxation” such as sleep and rest as an important strategy in managing their recovery. They also distracted themselves from postoperative pain by watching movies or television, playing video games, and listening to stories.

The caregivers verified the children's use of complementary strategies, and emphasized the importance of emotional support, closeness, positioning, and distraction during painful episodes. They described the use of distraction in terms of “focusing away the pain,” and spoke about how this was realized through calm activities such as listening to music, playing games, or watching television. One caregiver found it helpful to let the child sit up when she woke during the night.

#### 3.2.3 Compliance

Children's compliance was prominent in areas concerning activities and intake of analgesics. Despite the desire and ability to play and be physically active, they restricted themselves due to healthcare or caregiver instructions. The children also described how they took the analgesics even though they tasted terrible or were painful to swallow.

Caregivers’ compliance with the child's situation constituted allowing the child to choose their food, adapting the family's activities in line with the child's abilities, extending their sick leave, or testing multiple analgesics to find the most comfortable one for the child to take. The caregivers also adjusted the number of days of painkillers according to the child's need for pain relief, which meant they sometimes deviated from the healthcare instructions.

#### 3.2.4 Self‐reliance

Some caregivers expressed self‐reliance in dealing with their child's recovery. This was exemplified by a sense of security in making assessments and taking initiatives and decisions in challenging situations (eg, severe pain) or in cases where healthcare instructions were lacking or inconsistent. Some caregivers expressed uncertainty, ambivalence, or anxiety about the responsibility associated with their child's recovery. Expressions such as “hopelessness” and “helplessness” were used, and one caregiver even questioned her decision to let her child undergo the operation.

### 3.3 Impact on daily life

This category refers to the recovery period as an interruption in daily life, including the impact on basic functions and physical and psychological well‐being. The category is formed from three subcategories: Psychological and behavioral reactions, physical reactions, and impact on basic functions (Table 5).

#### 3.3.1 Physical reactions

The pain was the most strongly highlighted of all physical reactions during the postoperative period. The children clearly described how their pain was often worst in the morning and when they ate and swallowed. This pain was mainly localized in the throat, but many children also reported painful earache a few days after surgery. For some children, earache was described as the most painful and troublesome symptom. When describing pain, the children used the terms “burning,” “stinging,” and “strange,” and said it was like “scraping your knee, but in your throat.” Other physical symptoms described by the children were nausea, dizziness, and tiredness.

Many of the caregivers described situations with troublesome and often prolonged pain that was sometimes too much for the child to handle, using terms such as “panic,” “tortured,” and “extreme pain.” They also expressed confusion and frustration over the setback that occurred after a few postoperative days. Some described an absence of pain on the day of surgery followed by increased pain levels on the following days. In addition, they highlighted the children's fatigue, which manifested as a noticeably decreased activity level and an increased need for sleep during the daytime.

#### 3.3.2 Psychological and behavioral reactions

Children's emotions indicating fear during their recovery were prominent during the interviews. They explicitly described fear of pain and bleeding, and fear associated with the intake of tablets. Negative emotions around the loss of daily life, frustration over being physically inactive, and the sadness of not eating and playing as usual also arose during the interviews. The children expressed that their caregivers’ presence and emotional support were crucial during the recovery.

Psychological reactions described by caregivers included children's increased need for closeness, manifesting as a desire to be carried around, to sit in the caregiver's lap, and to sleep close by in a shared bed at night. They described the children's sleep as restless with frequent awakenings, which some caregivers said were “hysterical” and occurred without the child being aware. Terms such as “nightmares” and “night terrors” were frequently used to describe these new and unexpected situations. In terms of sadness, restlessness, depression, and anxiousness, the caregivers said their children were far from their ordinary selves during the recovery.

#### 3.3.3 Impact on basic functions

The children described how the postoperative recovery had an impact on their food intake, sleep, play, and verbal communication. Difficulties with eating and drinking were mentioned as a central concern that caused problems for both the children and their caregivers, and that resulted in the child's diet being mostly based on a certain type of soft food (ice cream, fruit purée, children's juice packs, yogurt, pasta, or cold soups).

Some children explained how the problems associated with the intake of certain food resulted in them avoiding eating and left them feeling hungry. The caregivers verified their children's descriptions of sparse food intake, and described how they tried several food options to find something suitable for the child to eat, and also adjusted the timing of meals to occur after the analgesics had taken effect.

The children generally reported getting a good night's sleep, but some remembered and described awakenings caused by pain. Moreover, they said they were unable to play as usual due to a lack of energy and due to exhortations from caregivers and healthcare not to exert themselves physically. They also described frustration over being forced to stay inside and rest. This was verified by the caregivers, who stated how they had to regularly remind the children to stay indoors and not physically exert themselves by running, jumping on the trampoline, and cycling in order to follow the healthcare instructions.

## 4 DISCUSSION

Based on interviews with children and caregivers, this study provides a unique insight into how recovery from tonsil surgery is experienced and managed by families in the home setting. Both children and caregivers reported the postoperative period to be an evident interruption in daily life, with an impact on physical and psychological well‐being. The results also indicate the families’ lack of preparedness for the multi‐faceted experiences encountered during the different postoperative phases. To bridge the discrepancy between the needs of the families and this lack of preparedness, the information provided by healthcare professionals needs to be broadened.

The pain was a highlighted concern, and some caregivers described the pain intensity as being too much for the child to handle. The pain was surrounded by a range of physical symptoms and behavioral changes, and had an impact on daily activities and the child's emotional state. In addition to suffering, uncontrolled postoperative pain is associated with an increased risk of developing short‐term and long‐term complications such as delayed behavioral and clinical recovery.^25^, ^26^, ^27^ This is an urgent issue to address. To be able to support the exposed families, all included healthcare professionals need to be aware of the difficulties the families are facing, and how to best facilitate their increased need for support even after being discharged from the healthcare setting.

In the absence of sufficient information from healthcare professionals, some families seek input and advice from family and friends.^28^ Our study shows that seeking answers in someone else's surgery on the tonsils could lead to new questions about differences in outcomes. Since many families also described conditions for which they were both unprepared and unaware, we suggest that the profession should provide families with a reliable description of the postoperative recovery that can be used as a reference point. This description should include information about recovery that may last as long as two weeks, with the possible presence of referred pain (earache), restlessness, disturbed sleep, nightmares, fatigue, nutrition difficulties and behavior changes in the child.

Reports of breakthrough pain and situations where the pain was difficult to manage were common in our interviews. The caregivers lacked any options other than ibuprofen and paracetamol to give when the pain was at its worst. The need for rescue analgesics was also identified in the quantitative data from this population.^4^ Opioids, particularly products containing codeine, have traditionally played a role in pain treatment after tonsillectomy.^29^, ^30^ However, opioid use carries a risk of respiratory depression, particularly in patients with obstructive sleep apnea,^29^, ^31^ and multiple regulatory authorities (*eg, the United States Food and Drug Administration*) warn against using codeine in children and contra‐indicate use following tonsil surgery.^32^, ^33^ This has left a gap in the treatment options.^29^ In some guidelines, clonidine has been suggested as an alternative to opioids since it has no effect on the respiratory drive.^34^ Future studies are needed regarding rescue medication for optimal pain management with minimal side effects.

The present results reveal multiple problems with analgesic administration, including the children's unwillingness to take analgesic medicine, poor and inconsistent instructions, the pharmacies not stocking the right strength or formulation, and difficulty converting formulation and route of administration. These issues are in line with the findings of a review^16^ which identified the main barriers to optimal postoperative analgesia at home to be inadequate administration, including parental and child factors, and inadequate prescription, including medication and system factors. To address these issues, the information and instructions must contain more than a fixed dosing schedule. In order to meet the child's preference, we suggest that the dosing schedule should be flexible in terms of treatment length, formulation, and route of administration. Families should be informed about the rationale for analgesic selection and potential side effects. An earlier study found that caregivers may not provide a night dose^4^ and so professionals need to also emphasize the importance of regular administration, even at night. In line with another previous study,^35^ our findings indicate that instructions to use a logbook and timer could facilitate the around‐the‐clock regimen. Caregivers should be encouraged to ask their child about pain, and to be aware of and sensitive to other signs of pain such as withdrawal, demand for contact, quietness, aggression and sleep disturbance. Families should receive both verbal and written contact information to allow them to contact healthcare staff for guidance if they cannot control the pain.

In line with previous studies,^36^, ^37^, ^38^ the families used different complementary strategies after tonsil surgery: comfort measures/emotional support, relaxation, distraction, and the application of cold. Support and education from healthcare professionals can facilitate children's and caregivers’ use of complementary methods after tonsil surgery.^39^, ^40^ Complementary methods are an essential part of the multimodal management of pain, and so we recommend that information about such strategies should always be included in the information given to the families.

The information regarding the risk of bleeding is another example of the importance of specific information. Both children and caregivers described an awareness of the risk for postoperative bleeding based on the information given. Some complications (eg, bleeding) can be considered more serious by healthcare providers, and hence are emphasized in the patient information.^41^ Information about bleeding is of utmost importance due to potentially severe and even fatal outcomes.^42^ However, our results indicate that patient information about physical activity restrictions linked to bleeding risk needs to be nuanced and exemplified to reduce unnecessary inactivity. The families also need to be informed that calm outdoor activity and fresh air influence the recovery in a positive way.

To summarize, the families in this study showed competence to manage their children's recovery after tonsil surgery at home, even though this period was challenging and suboptimal due to a lack of information and support from healthcare. Support from healthcare needs to involve more than just information about bleeding and a fixed analgesic schedule mediated by a single profession. Experiences and complications such as pain, possible setback after a few days, nightmares, and fatigue cannot be neglected when communicating with families. For this, a multidisciplinary team with complementary expertise and experience in tonsil surgery is crucial. To avoid providing inconsistent information, all professions involved must possess extensive knowledge and understanding of children's and caregivers’ perspectives of recovery after tonsil surgery, common postoperative conditions in the home setting, and pharmacological and non‐pharmacological methods. It is also essential not only to inform the families but also to ensure that they have understood and established readiness for action based on the information given. Since most of the pre and postoperative periods associated with tonsil surgery take place at home, the inclusion of new strategies for information and support is crucial. Here, the well‐established age‐differentiated websites tailored for children undergoing anesthesia and tonsil surgery (tonsilloperation.se/anaesthesiaweb.org) are useful resources.^43^, ^44^

### 4.1 Strengths and limitations

The strength of this study is that both children and caregivers from three hospitals participated. However, some limitations could be identified and need to be discussed. The first of these is the presence of possible selection bias. Although the selection was performed to capture variation regarding gender, age, and type of surgery method, recruitment took place within a cohort who had completed participation in a quantitative study.^4^ It can be assumed that families who had the organizational strength to complete and return the diary would also manage the recovery differently compared to non‐responders/non‐participants. Second, the potential for recall bias is present, as participants were interviewed weeks after surgery. However, supporting questions along with the pre‐filled diary were used to help the child remember and understand the interview context. The third is the challenge of capturing the child's opinion. It is essential that the child does not see the interview as “test questions” to see if they answer correctly, and does not respond to the questions with the aim of pleasing adults by producing the “right” answers. However, the interviewer used encouragement, and open‐ended questions to produce the richest possible data from the children.^45^

## 5 CONCLUSION

In order to optimize and support the recovery after tonsil surgery, it is crucial to obtain knowledge of children's and caregivers’ perspectives. The participants in this study described the experience of pain as significant, and often complex to manage. Besides pain, the postoperative period included several troublesome experiences for which neither the children nor the caregivers were informed or prepared. To prepare families for all the varied postoperative conditions and experiences, the information and support need to be broadened to cover more than bleeding and a fixed analgesic schedule. To increase families’ resilience, healthcare staff must provide reliable descriptions of common postoperative conditions, as well as individualized instructions including a range of options (pharmacological and complementary) to deal with these conditions. It is also essential not only to inform the families but also to ensure that they have understood and established readiness for action based on the information given. To achieve this goal, multidisciplinary teamwork is necessary. Extensive knowledge about children's and caregivers’ perspectives of recovery after tonsil surgery is a compulsory prerequisite for all involved. Further studies are required regarding rescue medication for optimal pain management with minimal side effects to address frequent breakthrough pain.

## CONFLICT OF INTEREST

None declared.

## APPENDIX 1 Interview guide

### Children

#### Opening question

*I’ve heard you were in the hospital having your tonsils removed. I’m trying to find out how children feel during the days after surgery, so I’m interested in hearing what it was like for you*.

Can you tell me about the days at home after your surgery?

​

#### Examples of follow‐up questions

When it happened, what did you do?
Can you tell me a little bit more about that?
How did you feel then?
You said pain. Can you tell me about a situation when you were in pain?
What was something you could do to make it better?
When the pain was at its worst, what could you do to make it better?
You said sore throat. What was it like to eat and drink with a sore throat?
Was there anything you could eat or drink to relieve the pain?
You were home from school. What did you do during the daytime?
You said you were taking medicine. How did it feel?
You said tired. What did you manage to do in the days after surgery?

​

#### Examples of supportive questions

What did you eat after returning home from the hospital?
What did you do the day after surgery?
If a friend of yours told you they were having the same surgery to remove their tonsils, and asked you what it would be like afterward, what would you tell them?
Would you have any tips to give your friend?

​

### Caregivers

#### Opening questions

*Now I want to hear how you thought the days were after the operation*.

Can you describe the days at home after your child's surgery?

​

#### Examples of follow‐up questions

When it happened, what did you do?
Can you describe such a situation?
How did you feel then?
Can you tell me a little bit more about that?
You said pain. Can you tell me about when your child was in pain?
How could you see that your child was in pain?
What was something you could do to relieve your child's pain?
When that happened, what could you do?
How was your child's diet?
How was your child's sleep?
What went better and what went badly when your child was trying to eat/drink?

​
